# Two boron-containing compounds affect the cellular viability of SH-SY5Y cells in an in vitro amyloid-beta toxicity model

**DOI:** 10.3906/biy-2001-22

**Published:** 2020-08-19

**Authors:** Mehmet OZANSOY, Mehmet Özgen ALTINTAŞ, Muzaffer Beyza OZANSOY, Necmeddin GÜNAY, Ertuğrul KILIÇ, Ülkan KILIÇ

**Affiliations:** 1 Department of Physiology, School of Medicine, Bahçeşehir University, İstanbul Turkey; 2 Regenerative and Restorative Medicine Research Center (REMER), İstanbul Medipol University, İstanbul Turkey; 3 Department of Physiology, School of Medicine, İstanbul Aydın University, İstanbul Turkey; 4 Department of Physiology, School of Medicine, İstanbul Medipol University, İstanbul Turkey; 5 Department of Medical Biology, School of Medicine, University of Health Sciences, İstanbul Turkey

**Keywords:** Boron, neuronal survival, amyloid-beta, Sirt1, GSK-3α/β

## Abstract

Boron is a naturally occurring trace element found in organic and inorganic complexes. Boron-containing compounds are required for living organisms for diverse metabolic functions, including nitrogen fixation in microorganisms, cell wall stability in plants, and bone and carbohydrate metabolism in animals. The number of studies about the effect of boron in biological model systems is very limited; so far, there has been no study on the correlation between boron and amyloid-beta toxicity. Here, we investigated the possible effects of 2 boron-containing compounds—sodium borate decahydrate and boric acid—against amyloid-beta toxicity. In our in vitro amyloid-beta toxicity model, we showed that these 2 compounds increase the survival of the SH-SY5Y cells. Furthermore, boron in these 2 forms increases the expression of Sirt1, which has protective functions against cellular stress. The compounds also change the expressions of GSK-3α/β; by doing so, boron may contribute to the stimulation of intracellular prosurvival pathways. This is the first experimental study indicating the prosurvival effect of boron in an amyloid-beta toxicity model.

## 1. Introduction

Boron is a naturally found trace element, according to the World Health Organization (1996). Boron has different isotopes and is not present in elemental form in nature (Howe, 1998). It usually forms organoboron complexes with oxygen and sodium. These organoboron complexes are biologically important for living organisms. In microorganisms, boron is required in many different metabolic functions as diverse as quorum sensing, nitrogen fixation, and antibiotic activity. In plants, it plays an essential role in cell wall stability and leaf expansion; its deficiency leads to dysfunction in expanding plant organs and cell adhesion due to the altered polymerization of the cytoskeleton and plasma membrane permeability. In the animal kingdom, inorganic borate compounds are transformed biologically and absorbed through mucosal surfaces; approximately 90% of the boron compounds given to animals is excreted in the form of boric acid. Recent studies have shown that boron has important functions in bone and carbohydrate metabolism, immunity, and the endocrinal system of mammals, including human beings. It is known that boron in the form of boric acid is found in the tissues and body fluids of humans and its deficiency causes a reduction in motor and cognitive abilities (Nielsen, 1997; Penland, 1998; Sutherland et al., 1998). In energy metabolism, there are at least 26 different enzymes whose activities are affected by boron, and boron containing compounds inhibit nicotinamide adenine dinucleotide (NAD+) (Strittmatter, 1964; Deitrich, 1967; Hunt et al., 1988; Aysan et al., 2011). It is well known that NAD+ and NADP+ are the 2 ribose-containing molecules with several functions in cellular energy metabolism; boron has a strong affinity to these cofactors (Hunt, 2012). Considering that oxidative damage is a molecular pathology in many different disorders, the role of boron in the energy metabolism of the cell gains additional importance.

Alzheimer’s disease (AD) is the most common progressive neurodegenerative disease worldwide (Blennow et al., 2006; Selkoe and Hardy, 2016). The two neuropathological hallmarks of the disease are intracytoplasmic protein inclusions, called neurofibrillary tangles (NFTs), and extracellular senile plaques, composed mainly of amyloid-beta (Aβ) protein fragments (Bennett et al., 2004; Götz et al., 2010). NFTs contain a hyperphosphorylated form of microtubule-associated protein tau in the form of paired helical filaments (PHFs), and these tau aggregates generate toxicity and drive the neuron to apoptosis (Götz et al., 2010). During the degenerative process of AD, the tau aggregates progressively accumulate in the soma of diseased neurons, dystrophic neurites, and neuropil threads; this NFT deposition leads to loss of synaptic function and neuronal death (Khan and Bloom, 2016). 

The other neuropathological feature of AD is extracellular amyloid-beta (Aβ) plaques. Aβ is primarily found in senile plaques and is generated by the sequential proteolytic cleavage of amyloid precursor protein (APP) through the action of secretases (Shankar et al., 2008; Selkoe and Hardy, 2016). Generated Aβ fragments (39–42 amino acids in length) are secreted. Normally, this APP processing is kept in a steady state, but disruption of the metabolic balance of Aβ processing causes the formation of toxic aggregates, which are linked to AD pathogenesis. Recent literature also proves that extracellular Aβ aggregates trigger the intracellular deposition of tau and contribute to the formation of NFTs (Sadigh-Eteghad et al., 2015). 

As boron and its compounds have effects on cellular energy metabolism, they could also have effects on neurodegeneration, in which oxidative stress and related pathologies have major importance.

Here we aim to investigate the possible effects of 2 boron-containing compounds in an in vitro amyloid-beta toxicity model. It is also widely known that sirtuin families of proteins play critical roles in the survival of cells under different stress conditions, and one of the family members is Sirt1, which is an NAD+-dependent histone deacetylase. Boron-containing compounds have effects on NAD+ metabolism; hence, Sirt1, having antisenescence and prosurvival effects, has been selected for this study (Gizem and Leonard, 2010, Aysan et al., 2011). Among the intracellular prosurvival pathways, Akt/GSK3-α/β is a central pathway; these 2 proteins were chosen in our experimental setting in order to elucidate the possible roles of 2 boron-containing compounds in cell survival.

## 2. Materials and methods

### 2.1. Establishing in vitro Aβ1–42 toxicity model

SH-SY5Y human neuroblastoma cell line was purchased (ATCC, USA) and they were cultured with Dulbecco’s Modified Eagles Medium (DMEM) containing 10% (v/v) heat-inactivated fetal bovine serum (Gibco, Gaithersburg, MD, USA) and 100U penicillin–streptomycin (Gibco). 

Cells were seeded in a 96-well plate at a density of 10,000cells/well; after 24 h, 4 different Aβ1-42 (Abcam, Cat No: 120301; Cambridge, UK) concentrations (1.25µM, 2.5 µM, 5 µM, and10 µM) were applied in order to find the effective toxic dose. Aβ1–42 was dissolved in 1% (v/v) NH4OH (Larson, 2016). Lactate dehydrogenase (LDH) assay (Roche, Cat No: 11644793001; Mannheim, Germany) was performed for cell viability measurements. Briefly, the working solution of the LDH assay was prepared according to the manufacturer’s instructions and was incubated in complete darkness for 15 min at room temperature. The medium in which our cells were cultured was put into a new 96-well plate in exactly the same order as the original plate. Next, 100 µL of LDH working solution was added to each well containing the cell culture medium to produce a final volume of 200 μL. At 492 nm, absorbance values in each well were measured in a microplate reader (Chromate Manager 4300, Awareness Technology, Palm City, FL, USA). 

### 2.2. Application of boron-containing compounds

Sodium borate decahydrate (SBD) (Sigma-Aldrich, Cat No: S9640, St. Louis, MO, USA) and boric acid (BA) (Sigma-Aldrich, Cat No: B6768) were dissolved in complete cell culture medium and filtered with 0.22-μm filters (99722, TPP, Trasadingen, Switzerland). SH-SY5Y cells were cultured in 96-well plates with 10,000 cells/well; after overnight incubation, the boron-containing compounds were added into the cells in 5, 10, 15, 20, 50, 100, and 200 μg/mL concentrations. After 72 h of incubation, the toxicity of the compounds on these cells was assessed by LDH analysis. With this analysis, the optimum nontoxic doses of the 2 compounds were determined.

### 2.3. Immunocytochemistry

SH-SY5Y cells were seeded into Petri dishes at a density of 50,000 cells/dish. After 24 h, Aβ1–42 (10 µM) was applied and cells were incubated for 48 h. Using primary Aβ1–42 antibody (Santa Cruz Biotechnology, Cat No: sc-28365, Santa Cruz, CA, USA), amyloid-beta aggregates were visualized fluorescently using laser confocal microscopy. Cell nuclei were visualized with DAPI. Petri dishes in which SH-SY5Y cells were cultured without Aβ1–42 were used as negative controls. All microscopy experiments were conducted at least twice with different Aβ preparations.

### 2.4. Western blotting

At the end of the incubations, cells were detached with 1XPBS (Phosphate buffered saline, Gibco). After pelleting the cells by centrifugation at 3000 rpm for 10 min at +4 °C, lysis buffer (1M Tris-HCl, 5M NaCl, Triton-X-100, 0.5 M EDTA) containing a protease/phosphatase inhibitor cocktail (Cell Signaling Technology, Cat No: 5872, Danvers, MA, USA) was added to the cells. Samples were centrifuged at 14,000 rpm for 15 min at +4 °C and supernatants were collected. Protein concentrations were determined spectrophotometrically (Implen, München, Germany). 

Forty µg of each protein sample from cell lysates were separated by 4%–12% NuPAGE electrophoresis system and samples were transferred to polyvinylidene fluoride membranes (PVDF) using an iBlot Dry Blotting System (Invitrogen, Carlsbad, CA, USA). Membranes were blocked in 5% nonfat milk in 50mM Tris-buffered saline containing 0.1% Tween-20 for 1 h at room temperature, then washed in Tris-buffered saline containing 0.1% Tween-20. The membranes were treated with the following primary antibodies overnight at +4 °C: phospho-Akt (Cell Signaling Technology, Cat No: 4051), total Akt (Cell Signaling Technology, Cat No: 2920), phospho-GSK3β (Cell Signaling Technology, Cat No: 8566), total GSK3β (Cell Signaling Technology, Cat No: 9832), phospho-Tau (Cell Signaling Technology, Cat No: 12885) and Sirt1 (Cell Signaling Technology, Cat No: 9475). Primary antibodies were diluted to 1:1000 in Tris-buffered saline containing 0.1% Tween-20. On the second day, the membranes were washed and further incubated in blocking solution with peroxidase-conjugated–secondary antibody (Cell Signaling, Cat No: 7074S) for 1 h at room temperature. 

All blots were performed at least 3 times and revealed using a ECL-Advanced Western Blotting Detection Kit according to the manufacturer’s protocol (Amersham BioSciences, Cat No: RPN2232, Little Chalfont, UK). Proteins were visualized with a Bio-Rad ChemiDoc XRS System (Bio-Rad Laboratories Inc., Philadelphia, PA, USA) and analyzed densitometrically with ImageJ software. 

### 2.5. Statistical analysis

Data were statistically evaluated with one-way ANOVA. A P value of less than 0.05 was regarded as being statistically significant. For statistical data comparisons, a standard software package (SPSS 18 for Windows; SPSS Inc., Chicago, IL, USA) was used. Data were statistically analyzed by using repeated-measures analysis of variance (ANOVA), followed by the Tukey post-hoc test. Values are given as mean and standard deviation of the mean (SD). 

## 3. Results

### 3.1. The cell viability assays

**Figure 1:**
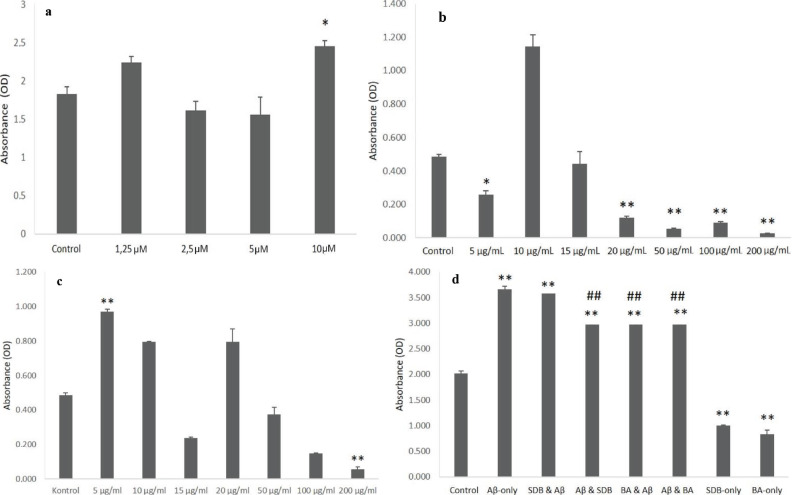
LDH analyses of different concentrations of (a) Aβ_1-42_ (Elibol, 2019) , (b) Boric Acid (BA), (c) Sodium Borate Decahydrate (SBD), and (d) effects of 200 μg/mL of BA and SBD on the viability of cells when 10 μM Aβ_1-42_ applied. *: P < 0.05; **: P < 0.01 against the control group; ##: P < 0.01 against Aβ-only group.

In order to determine the most effective toxic dose of Aβ on the SH-SY5Y cells, 4 different concentrations of Aβ_1-42_ (1.25 µM, 2.5 µM, 5 µM, and10 µM) were tested by LDH analysis. Cell viability data showed that the 10 µM Aβ_1-42_ dose was the most effective toxic dose for these cells (P < 0.05, Figure 1a). The possible toxic effects of the 2 boron-containing compounds (sodium borate decahydrate: SBD, and boric acid: BA) were also investigated by LDH analysis for 5, 10, 15, 20, 50, and 200 µg/mL concentrations; the highest nontoxic concentrations of both compounds, i.e. 200 µg/mL, were selected for our study (P < 0.01, Figure 1b and 1c).

After determining the most effective toxic dose of Aβ_1-42_ and nontoxic doses of SBD and BA, the effects of these 2 compounds on amyloid-beta toxicity were studied. In order to assess their possible effects, SBD and BA were applied 24 h before the Aβ_1-42_ treatment; they were also applied 24 h after Aβ_1-42_ treatment. After 48 h of incubation, cell viability was measured by LDH analysis and it was shown that pre- and posttreatment of BA led to significant increase in cell viability when compared with the Aβ-only group (P < 0.01, Figure 1c). SBD posttreatment also showed a significant rise in cell survival when compared with the Aβ-only group (P < 0.01, Figure 1d).

### 3.2. Cellular imaging

**Figure 2:**
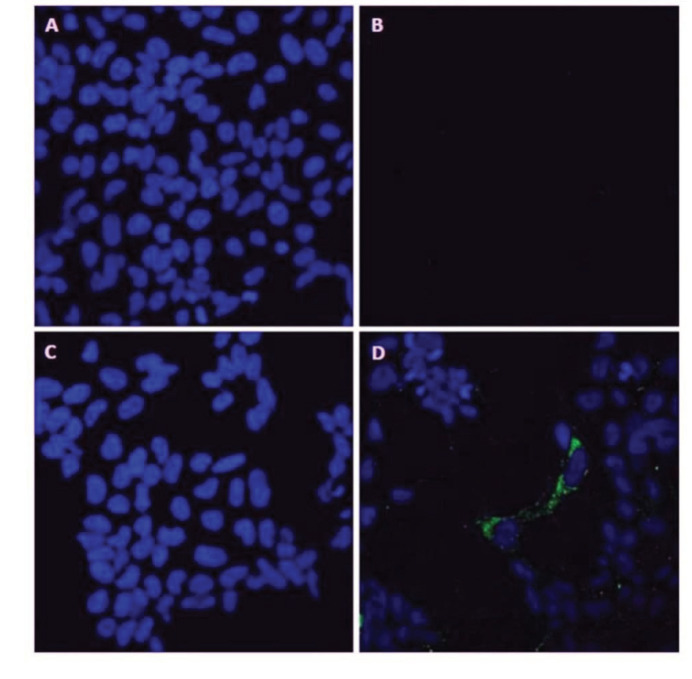
Immunocytochemical imaging. (a) shows the cell nuclei of the control sample without Aβ, (b) exhibits the control sample without Aβ incubated with anti-Aβ antibody, (c) shows the cell nuclei of the sample where 10 μM Aβ has been applied, (d) the sample where 10 μM Aβ has been applied and incubated with anti-Aβ antibody. The green fluorescence shows the amyloid beta aggregates inside the inside the SH-SY5Y cells where 10 μM Aβ has been applied.

After SH-SY5Y cells were cultured, 10 µM Aβ_1-42_ was applied. At the end of 48 h of Aβ_1-42_ incubation, cells were fixed with 4% paraformaldehyde. After permeabilization, anti-Aβ antibody was used. Control samples were treated with secondary antibody only. Laser confocal microscopy revealed that amyloid-beta was internalized by the cells (Figure 2).

### 3.3. Protein expression analyses

**Figure 3:**
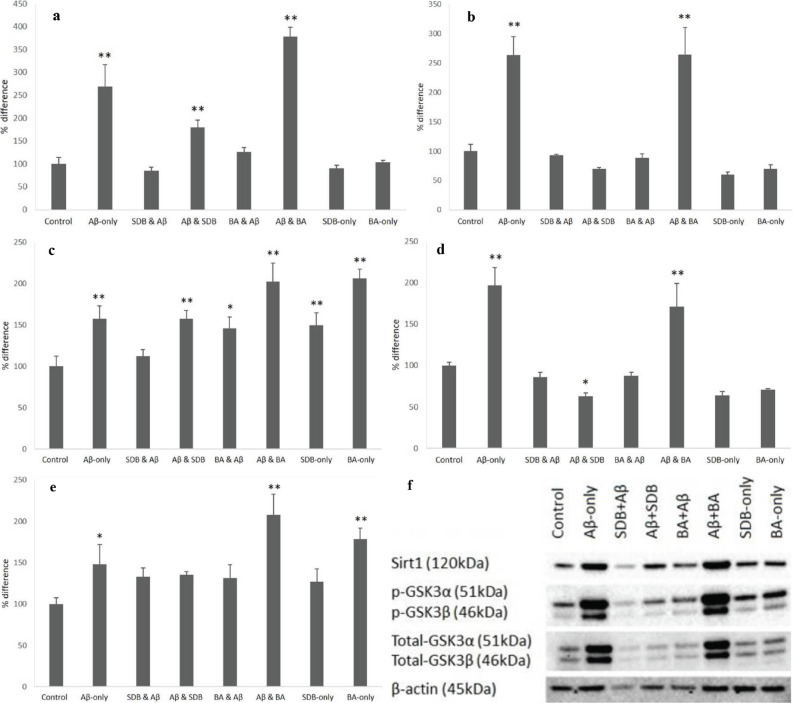
Quantification of western blotting data and the image of the blot. (a) the expression of Sirt1, (b) the expression of total GSK-3β, (c) the expression of p-GSK-3β, (d) the expression of total GSK-3α, (e) the expression of p-GSK-3α, (f) western blot membrane. SBD: Sodium borate decahydrate; BA: Boric acid; *: P < 0.05, **: P < 0.01.

At the end of the incubation, cells were lysed as was mentioned above and Sirt1, total, and phosphorylated forms of GSK-3α/β and Akt were visualized and quantified. Band intensities were normalized with corresponding β-Actin bands (Figure 3a). 

Although Aβ_1-42_ application increased Sirt1 expression significantly, posttreatment of BA increased Sirt1 expression more strongly (P < 0.01, Figure3b). On the other hand, posttreatment of SBD led to a statistically significant decrease in Sirt1 expression (P < 0.01, Figure 3b). Other experimental groups did not show any statistically significant changes in Sirt1 expression level.

A similar pattern was observed in total GSK-3α levels. Aβ_1-42_ application and posttreatment of BA both led to statistically significant elevations, but the amount of the elevation was more prominent in the latter (P < 0.01, Figure 3c). When phosphorylated GSK-3α (pGSK-3α) level was assessed, its expression was found to be increased in all experimental groups except for the SBD pretreatment group (P < 0.01, Figure 3d).

When total GSK-3β levels were analyzed, there were significant increases in the Aβ-only group and the BA posttreatment group (P < 0.01, Figure 3e). When pGSK-3β levels were studied, it was found that significant elevations were observed in Aβ-only, SBD-only, and BA posttreatment groups (P < 0.01, Figure 3f).

There were no statistically significant differences in any experimental groups when Akt and pAkt were analyzed (data not shown).

## 4. Discussion

Boron is a naturally occurring mineral mostly found in water-soluble forms. Boron-containing compounds are ingested through a normal daily diet; because of their high solubility and turn-over rate, they are excreted rapidly (Howe, 1998). Although it has a high clearance rate, boron has certain cellular and histological effects on living organisms. It is already known that boron and boron-containing compounds have regulatory effects on NAD+ and NADP+ metabolism; through these effects they may have roles in cellular energy metabolism (Nielsen, 1997; Howe, 1998). 

Here, we have established an in vitro toxicity model for investigating the possible effects of 2 boron-containing compounds. In this model, Aβ_1-42_ was chosen to confer toxicity, because it is clear from the relevant scientific literature that amyloid-beta aggregates in the brain increase in amount with aging, and with several neurodegenerative disorders including Alzheimer’s disease. These aggregates eventually lead to neuronal toxicity and death (Shankar et al., 2008; Sadigh-Eteghad et al., 2015; Selkoe and Hardy, 2016).

In the present study, we have showed that both sodium borate decahydrate (SBD) and boric acid (BA) have viability-supporting effects in the amyloid-beta toxicity model when they were used at 200 µg/mL doses. The cell viability data of our study show that 10 µg/mL BA is more toxic than the other concentrations, and 5 µg/mL BA is less toxic than 10 and 15 µg/mL concentrations. When the effects of SBD are examined, a different picture emerges. For example, 5 µg/mL SBD seems to be the most toxic dose, and while 15 µg/mL of SBD exhibits less toxicity than 10 and 20 µg/mL concentrations, toxicity starts to increase again at 20 µg/mL, and then begins to drop. All of these findings about the toxicity of BA and SBD for different concentrations clearly indicate that the SH-SY5Y cells respond to these compounds nonlinearly. Nonlinearity to external insults or effects is one of the common characteristics of all living systems, from unicellular organisms to mammalians. This nonlinear response to BA and SBD in the context of toxicity needs to be investigated extensively, since the possible biological effects of boron and/or boron-containing compounds have only been studied in a limited fashion.

The implemented protein analyses might indicate the possible mechanistic links between the prosurvival effects of SBD and BA and intracellular signaling pathways. First of all, amyloid-beta application by itself increases Sirt1 expression, and this would probably depend on the function of Sirt1 in the cell. It has been revealed that Sirt1, as a NAD+-dependent histone deacetylase, has a protective role against oxidative stress (Rahman and Islam, 2011). It is also known that Sirt1 expression increases in Alzheimer’s disease and several other neurodegenerative disorders in order to give protection against rising neuronal stress (Kim et al., 2007; Michan and Sinclair, 2007; Gizem and Leonard, 2010). Our data show that when Aβ_1-42_ is applied to the SH-S5Y5 cells, Sirt1 expression is stimulated, and more importantly, boric acid (BA) posttreatment after Aβ_1-42_ application increases Sirt1 expression more than that of the Aβ-only group (Figure 3a). This finding indicates that BA posttreatment supports the protection against Aβ_1-42_ toxicity and the protective mechanism could be through by rising Sirt1 expression. 

The analysis of GSK-3α expression shows that total GSK-3α expression increased in the Aβ-only group and the posttreatment of BA group (Figure 3c). In addition to that, the phosphorylated form of GSK-3α also increased in all experimental groups except for the SBD pretreatment group (Figure 3d). It is clear from the other findings that GSK-3α plays a role in amyloid-beta precursor protein (APP) cleavage and processing.

We also studied the GSK-3α/β/Akt prosurvival pathway to determine if it is involved in the survival-supporting effects of boron-containing compounds. The data show that the amyloid-beta application and BA posttreatment groups both had increased total GSK-3β expression, and its phosphorylated form also increased in these same experimental groups (Figures 3e and 3f). Phosphorylated GSK-3β expression was found to be increased in the SBD-only group as well (Figure 3f). These findings about GSK-3β expression clearly reveal that intracellular prosurvival pathways are activated; however, this activation of GSK-3β could not be linked to the stimulation of Akt, because neither the total Akt expression nor its phosphorylated form were found to be increased (data not shown). 

Taken together, all of these findings indicate that SBD and BA have viability-increasing effects against amyloid-beta toxicity, and these effects may depend on the stimulation of GSK-3α/β expression. It may also be necessary to analyze this effect further, because changes seen in GSK-3α/β expression could not lead to Akt activation. It is also important to note that Sirt1 might also be involved in the protective effects of boron-containing compounds, especially boric acid. To the best of our knowledge, this is the first study investigating the effect of boron on amyloid-beta toxicity in an in vitro cell culture setting.
